# Supplementary material for: Mycobacterial HelD is a nucleic acids-clearing factor for RNA polymerase
Source: Nat Commun. 2020 Dec 18;11:6419. doi: 10.1038/s41467-020-20158-4 (PMC7749160; doi:10.1038/s41467-020-20158-4)
Supplement: Supplementary file 1 — Supplementary information [file 41467_2020_20158_MOESM1_ESM.pdf]

Supplementary information to

Mycobacterial HelD is a nucleic acids-clearing factor for RNA polymerase

Tomáš Kouba[1*#], Tomáš Koval'[2*], Petra Sudzinová[3*], Jiří Pospíšil[3], Barbora Brezovská[3], Jarmila Hnilicová[3], Hana Šanderová[3], Martina Janoušková[3], Michaela Šiková[3], Petr Halada[3], Michal Sýkora[4], Ivan Barvík[5], Jiří Nováček[6], Mária Trundová[2], Jarmila Dušková[2], Tereza Skálová[2], URee Chon[7], Katsuhiko S. Murakami[7], Jan Dohnálek[2#], Libor Krásný[3#]

[1] EMBL Grenoble, 71 Avenue des Martyrs, France

[2] Institute of Biotechnology of the Czech Academy of Sciences, Centre BIOCEV, Průmyslová 595, 252 50 Vestec, Czech Republic

[3] Institute of Microbiology of the Czech Academy of Sciences, Prague, Czech Republic

[4] Institute of Molecular Genetics of the Czech Academy of Sciences, Prague, Czech Republic

[5] Charles University, Faculty of Mathematics and Physics, Institute of Physics, Prague, Czech Republic

[6] CEITEC, Masaryk University, Brno, Czech Republic

[7] Department of Biochemistry and Molecular Biology, The Center for RNA Molecular Biology, Pennsylvania State University, University Park, PA 16802, USA

*These authors contributed equally to this work

#Corresponding authors: tkouba@embl.fr, dohnalek@ibt.cas.cz, krasny@biomed.cas.cz

This file includes:

Supplementary Figures 1 to 14

Supplementary Tables 1 to 7

Supplementary References

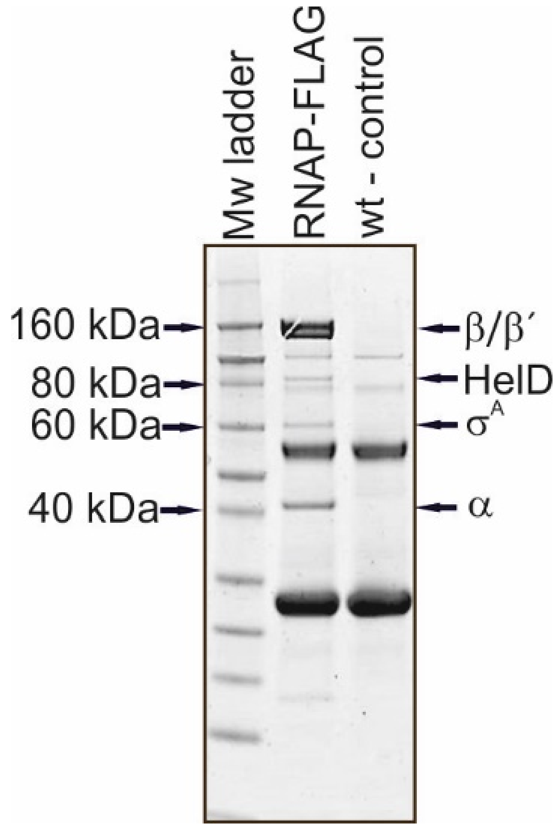

Supplementary Figure 1: *Msm* HelD is in complex with RNAP.

SDS-PAGE of IPs of RNAP-FLAG from *Msm* (RNAP-FLAG, strain LK1468; wt, strain LK865). The gel shows boiled ANTI-FLAG M2 agarose with bound proteins. The identities of the pulled-down proteins are indicated with arrows (determined by mass spectrometry). Wt – control, a strain without any FLAG-fusion. The experiment was performed 3x (biological replicates) with the same result. Mw, molecular weight marker. The two prominent un-marked bands correspond to heavy and light antibody chains, respectively.

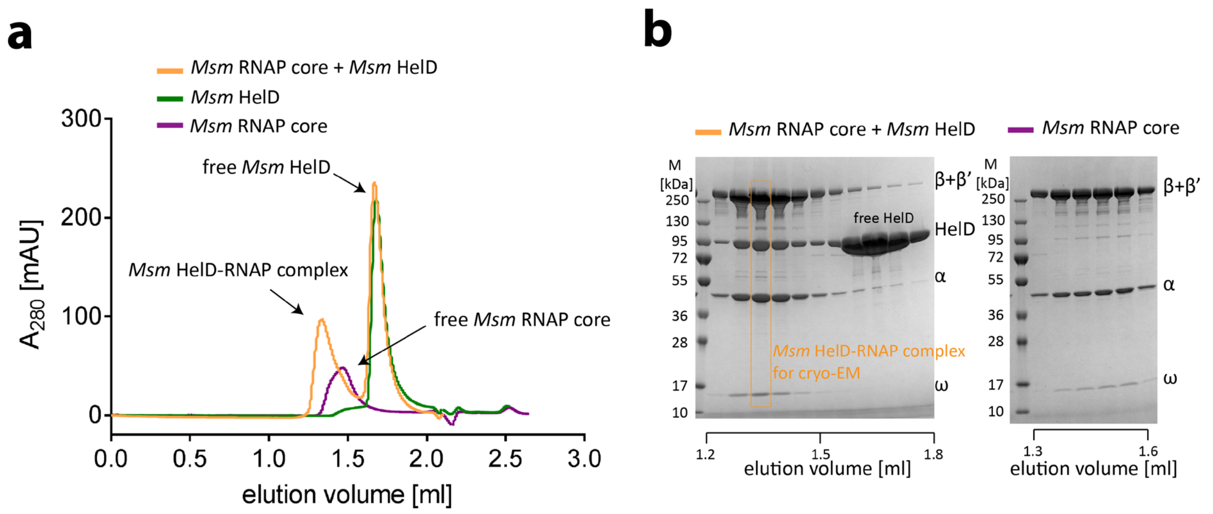

Supplementary Figure 2: Reconstitution of *Msm* HelD-RNAP complex.

a, Size-exclusion chromatography (SEC) analysis of RNAP core alone (purple line) and HelD protein alone (green line). SEC analysis of protein sample after reconstitution of RNAP core with HelD protein at a 1:3 ratio (yellow line). The first yellow peak (from left) is the *Msm* HelD-RNAP complex, the second yellow peak is excess of free HelD protein. The data were analysed and the graphics created with GraphPad Prism 7.02.

b, SDS-PAGE analysis of the *Msm* HelD-RNAP complex and the *Msm* RNAP core. 40 µg protein samples of fractions of *Msm* HelD-RNAP complex and RNAP core alone were loaded onto analytical SDS-PAGE. Fractions are indicated by the elution volume. The first lane contains the molecular weight marker.

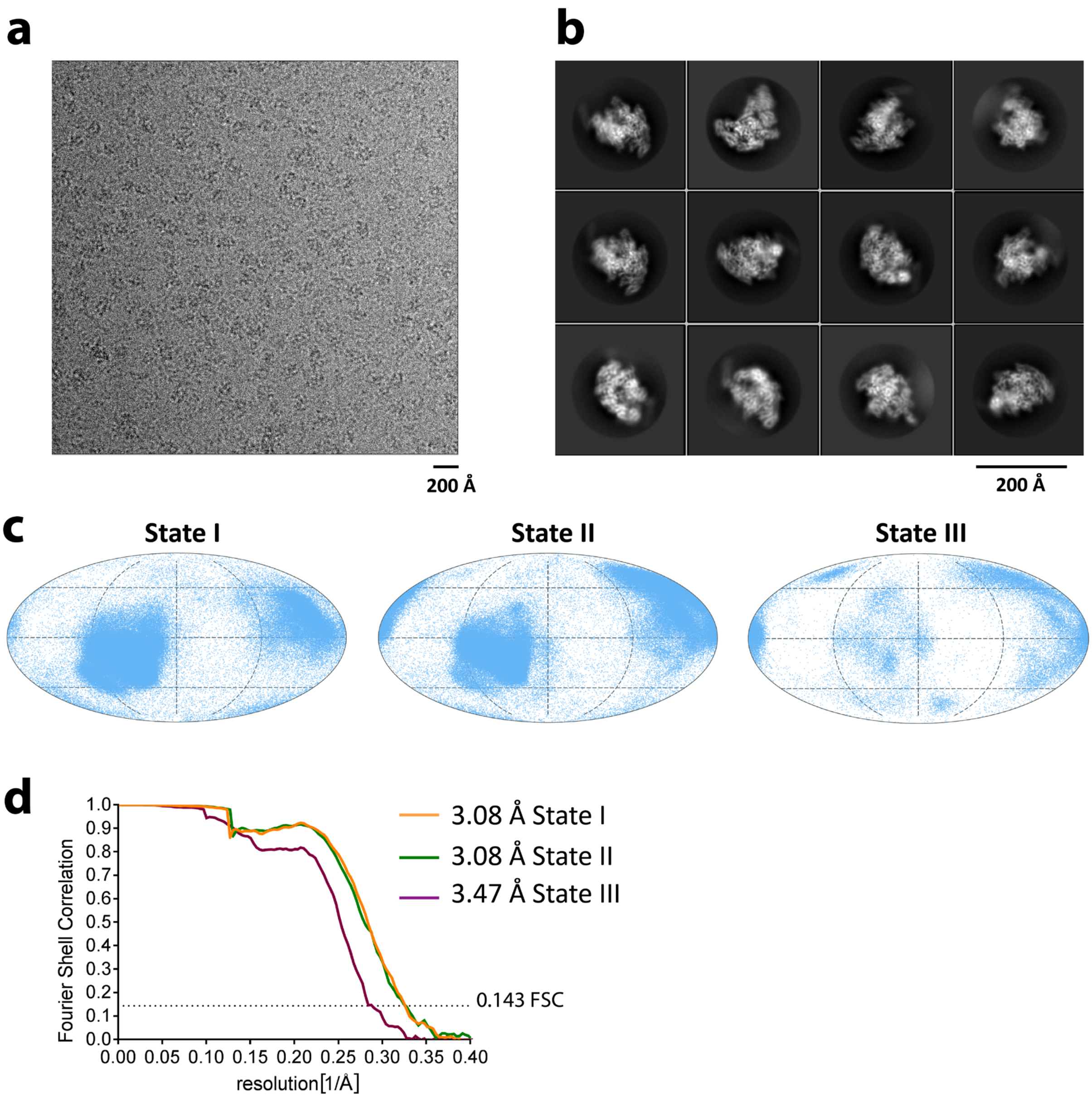

Supplementary Figure 3: Cryogenic electron microscopy of *Msm* HelD-RNAP complex.

a, Representative micrograph of *Msm* HelD-RNAP complex in free-standing ice after MotionCor2[1] correction at defocus of ~2.5 µm.

b, 2D-class averages of the *Msm* HelD-RNAP complex.

c, Angular distribution for particle projections of the *Msm* HelD-RNAP complex State I, II and III respectively, visualized on a globe-like plane. The data were analysed and the graphics created with cryoEF[2].

d, Fourier shell correlation (FSC) curves for *Msm* HelD-RNAP complex State I (yellow), II (green) and III (purple), respectively. The plot of the FSC between two independently refined half-maps shows the overall resolution of the two maps as indicated by the gold standard FSC 0.143 cut-off criteria[3]. The data were analysed and the graphics created with GraphPad Prism 7.02.

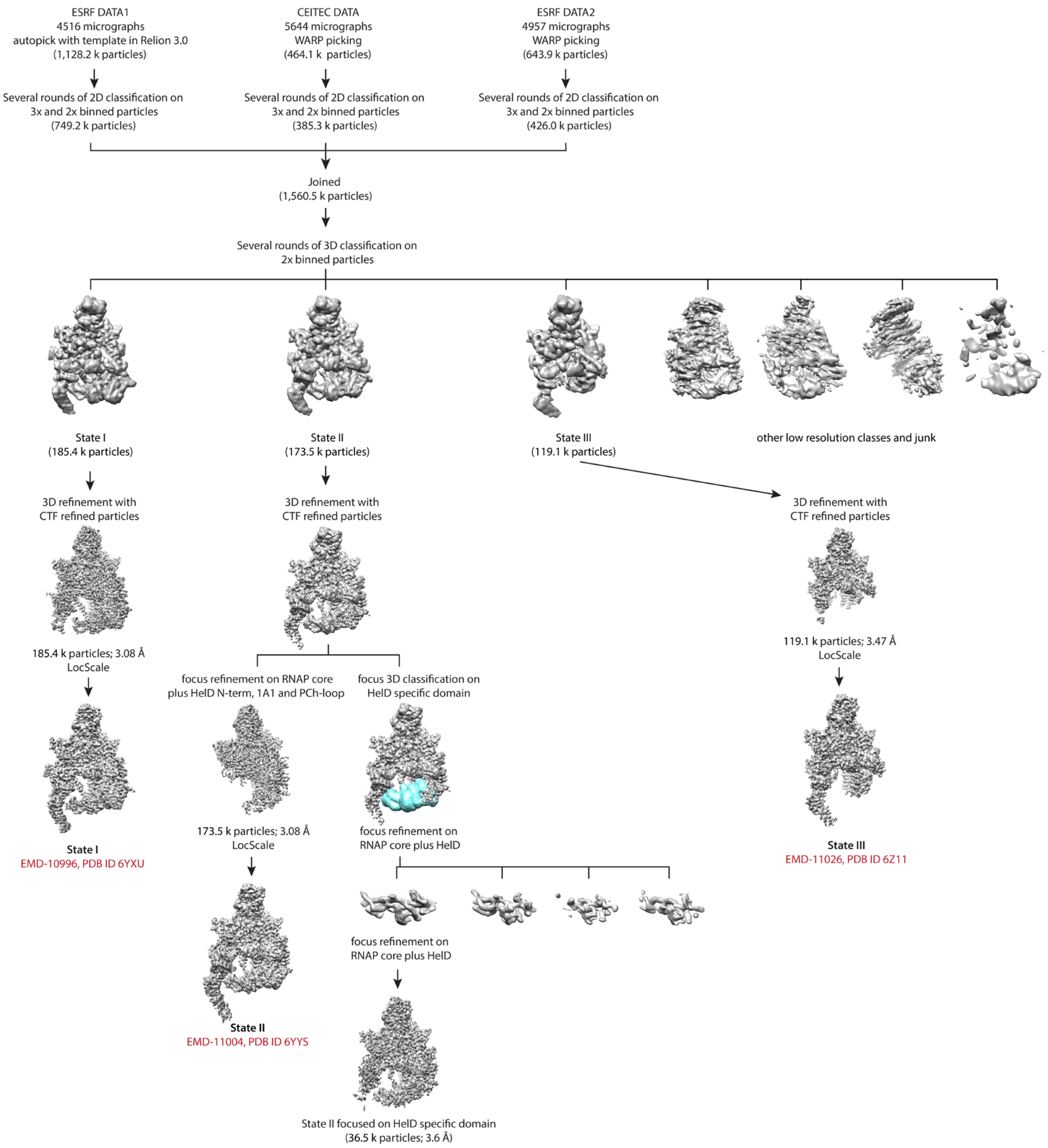

Supplementary Figure 4: Cryo-EM data 3D classification and refinement scheme.

Summary of the cryo-EM 3D classification and refinement scheme of the *Msm* HelD-RNAP complex. Initially, three different datasets were processed individually to the level of 2D classification. 2D classes with well-defined secondary structure features were merged (1,560.5k particles). The merged particles were classified into ten 3D classes with angular assignment. Incomplete, low resolution, and damaged particle classes were excluded from further data analyses. The three most prominent 3D classes of the *Msm* HelD-RNAP complex were refined, and subsequently filtered by LocScale[4], corresponding to State I, II and III. The State II class was focus-refined around the region of the RNAP core and the HelD N-terminal and 1A domain and PCh-loop. In parallel, a round of focus classification was performed on the region of the HelD 1A and HelD-specific domains using corresponding mask (cyan) in order to get a better defined map for model building of the latter region. Atomic resolution cryo-EM maps were refined and post-processed with their respective masks in RELION 3.0[5,6].

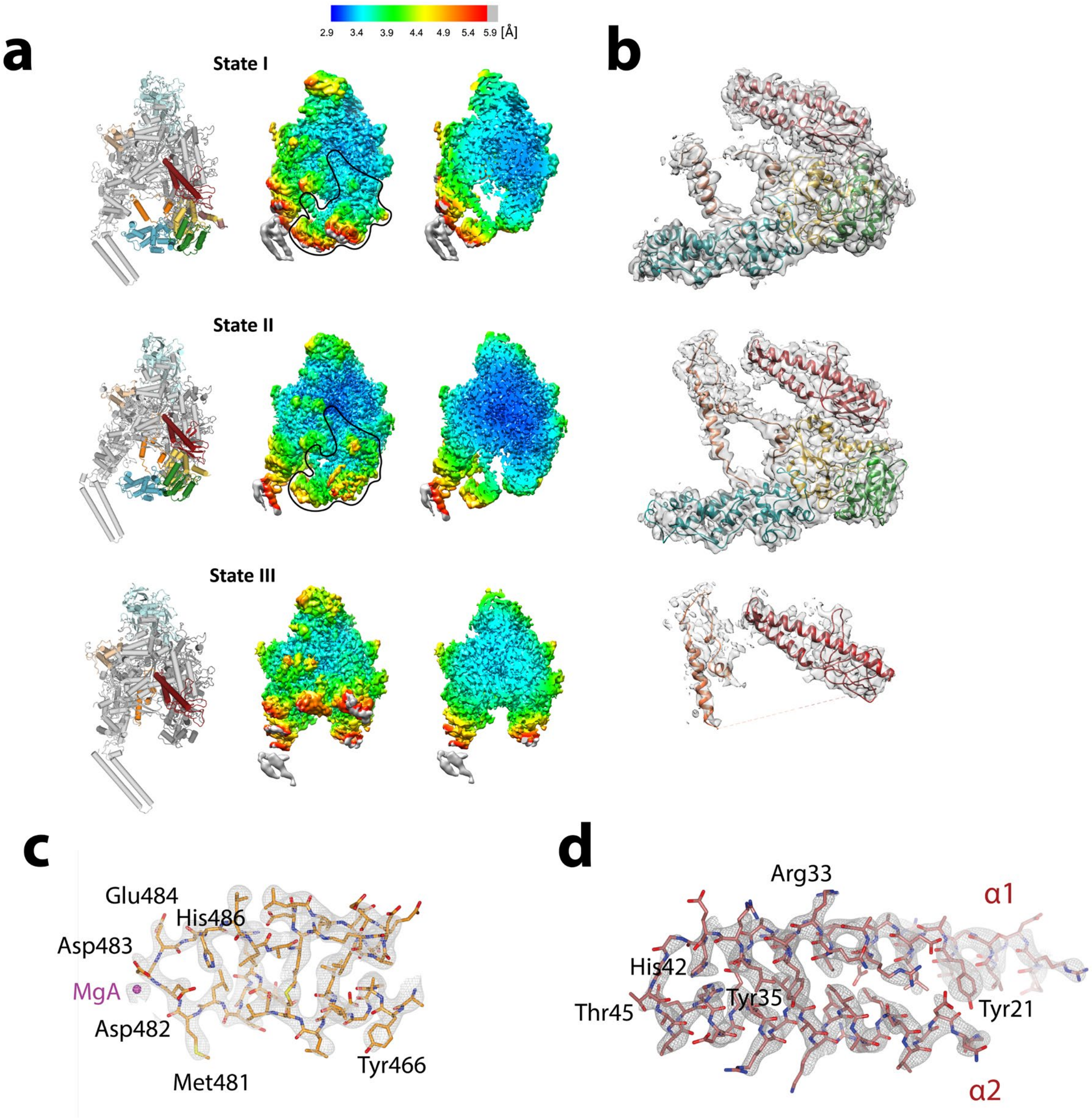

Supplementary Figure 5: Local resolution and cryo-EM density maps of the *Msm* HelD-RNAP complexes.

a, Cylinder model (**left**) and distribution of local resolution of the *Msm* HelD-RNAP State I, II and III, respectively. Surface (**middle**) and slice (**right**) representation. The black line in the middle panels delineates HelD in State I or II. Maps are colored according to the local resolution calculated within the RELION software package. Resolution is as indicated in the color bar. Graphics created with Pymol (Schrödinger, Inc.) and Chimera[7].

b, LocScale filtered cryo-EM density map for the *Msm* HelD protein in State I, II and III, respectively. Color coded as in Figure 1e. Graphics created with Chimera[7].

c, LocScale filtered cryo-EM density for the HelD PCh-loop tip, MgA is shown as magenta sphere. Carved with a 1.75 Å clip radius around the atomic model in CCP4mg[8].

d, LocScale filtered cryo-EM density for the N-terminal CC-domain of HelD carved with a 1.75 Å clip radius around the atomic model in CCP4mg[8].

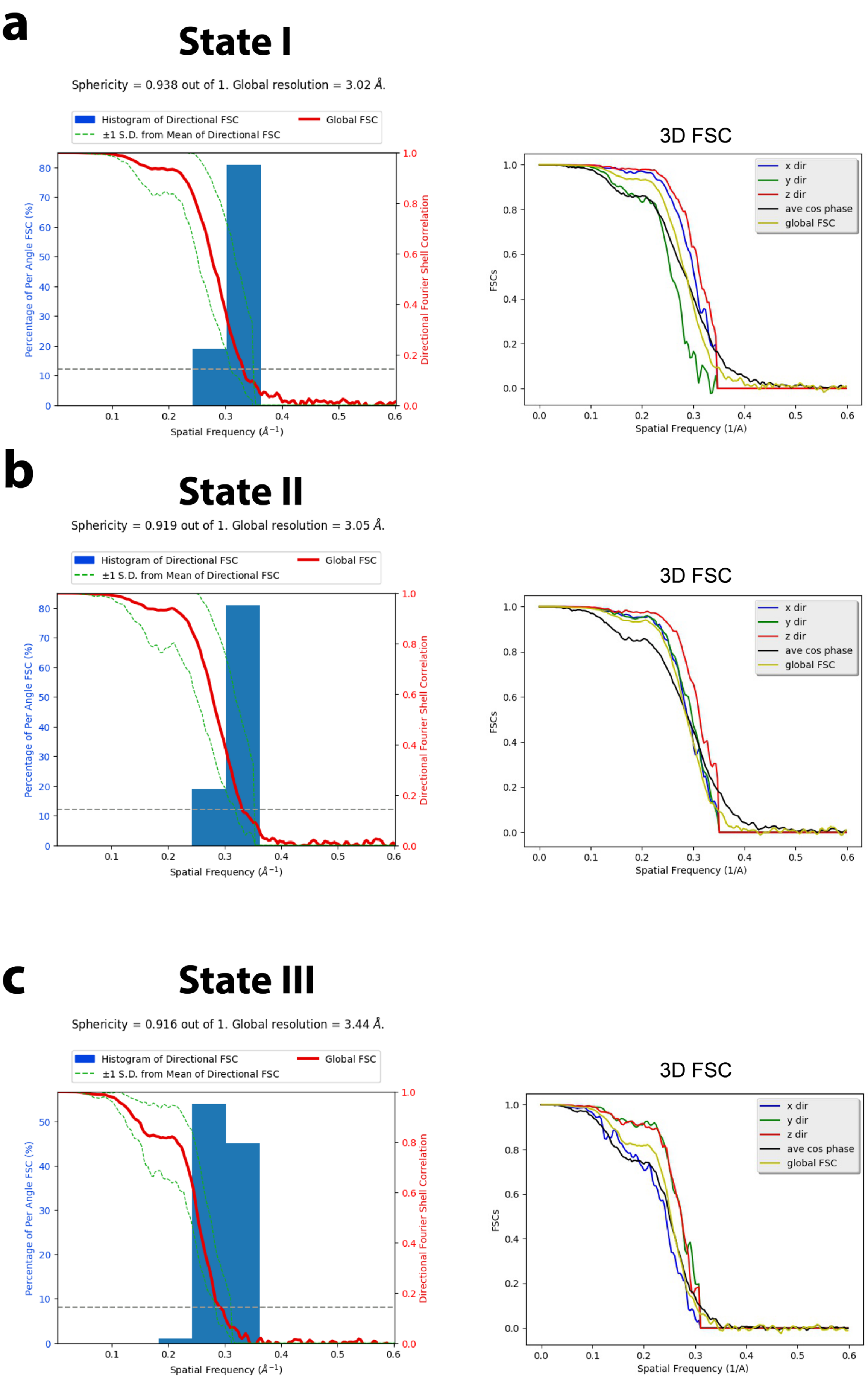
a
State I
Sphericity = 0.938 out of 1. Global resolution = 3.02 Å.
Histogram of Directional FSC
Global FSC
±1 S.D. from Mean of Directional FSC
Percentage of Per Angle FSC (%)
Directional Fourier Shell Correlation
Spatial Frequency (Å⁻¹)
3D FSC
x dir
y dir
z dir
ave cos phase
global FSC
FSCs
Spatial Frequency (1/A)
b
State II
Sphericity = 0.919 out of 1. Global resolution = 3.05 Å.
Histogram of Directional FSC
Global FSC
±1 S.D. from Mean of Directional FSC
Percentage of Per Angle FSC (%)
Directional Fourier Shell Correlation
Spatial Frequency (Å⁻¹)
3D FSC
x dir
y dir
z dir
ave cos phase
global FSC
FSCs
Spatial Frequency (1/A)
c
State III
Sphericity = 0.916 out of 1. Global resolution = 3.44 Å.
Histogram of Directional FSC
Global FSC
±1 S.D. from Mean of Directional FSC
Percentage of Per Angle FSC (%)
Directional Fourier Shell Correlation
Spatial Frequency (Å⁻¹)
3D FSC
x dir
y dir
z dir
ave cos phase
global FSC
FSCs
Spatial Frequency (1/A)

Supplementary Figure 6: 3D FSC analysis of HelD-RNAP complexes cryo-EM maps.

a, **b**, **c** Directional FSC analysis[9] (right) and 3D FSC analysis[9] (left) of HelD-RNAP in State I, II, and III, respectively. (**right**) Plots of the global half-map FSC (solid red line, right axis) together with the spread of directional resolution values defined by ±1σ from the mean (area encompassed by dotted green lines) and a histogram of Directional FSC (blue bars, left axis). (**left**) Directional FSC analysis in x (blue), y (green) and z (red) direction compared to the global (yellow) FSC analysis. The analysis was performed with the 3DFSC server v. 3.0[9].

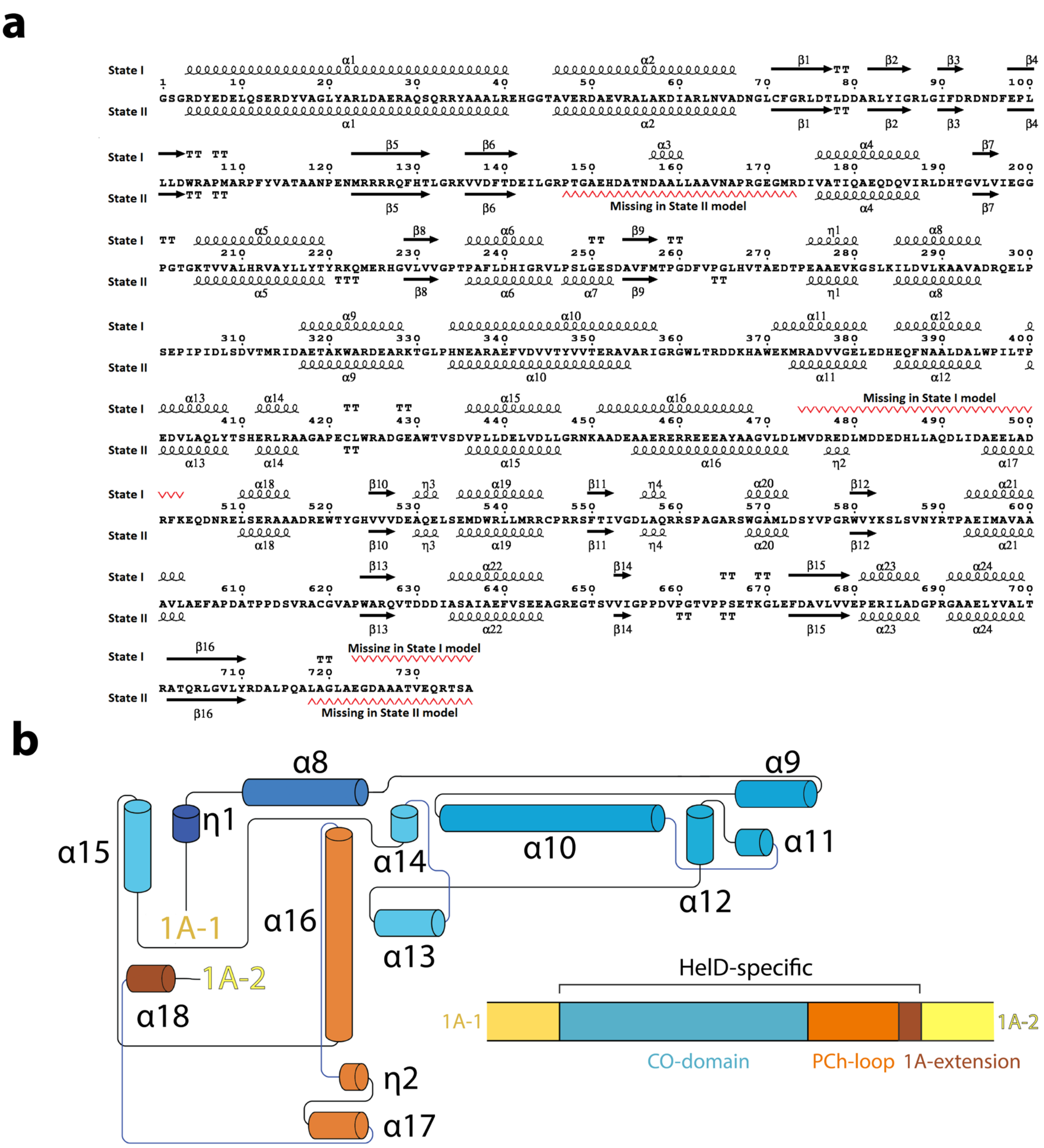

Supplementary Figure 7: Secondary structure assignment of HelD protein.

a, State I (**top**) and State II (**bottom**) secondary structure elements marked along the *Msm* HelD amino acid sequence. Some regions (red marking) are not folded in one or the other State, α7 exists in State II only, α16 has a shifted register. The graphics was created using ESPript 3.0[10].

b, Topology of the new fold of the HelD-specific domain (no structural homolog identified). The graphics was created using PDBsum server[11].

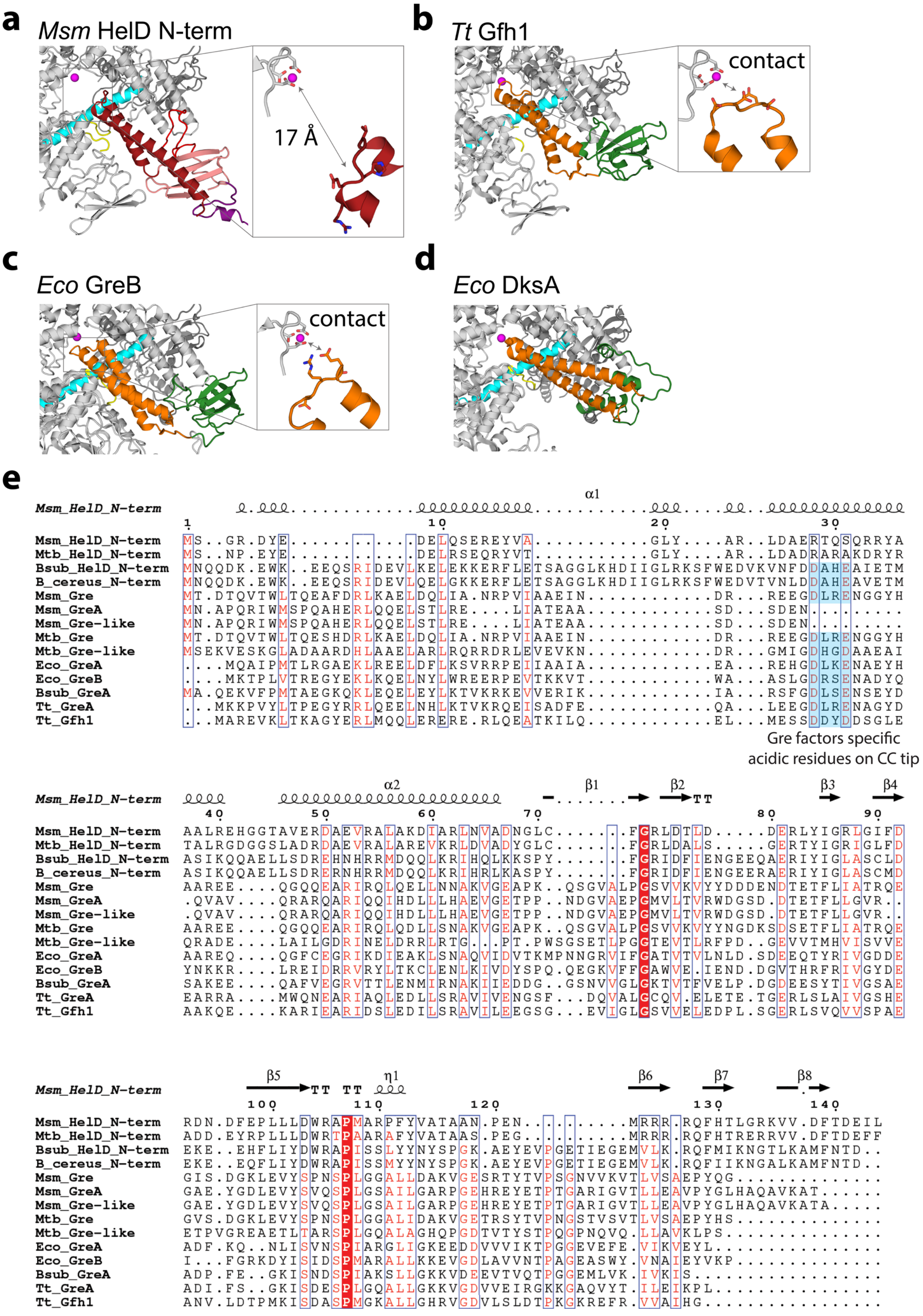
a
Msm HelD N-term
17 Å
b
Tt Gfh1
contact
c
Eco GreB
contact
d
Eco DksA
e
Gre factors specific
acidic residues on CC tip

Supplementary Figure 8: Structural comparison of HelD and Gre-like transcription factors

a, **b**, **c**, and **d** Structural comparisons of (**a**) *Msm* HelD N-terminal domain and Gre-like transcription factors. HelD anchors into the RNAP secondary channel similarly to (**b**) *Tt* Gfh1 (PDB ID 3AOH) and (**c**) *Eco* GreB (PDB ID 6RI7) N-terminal CC (orange) and globular (green) domains. However, in contrast to GreB and Gfh1 CC domains, the tip of HelD NCC-domain does not reach to the AS (insets, MgA as magenta sphere). (**d**) *Eco* DksA interacts with the RNAP secondary channel in a similar fashion (PDB ID 5W1T). Graphics created with Pymol (Schrödinger, Inc.).

e, Sequence alignment of HelD homologs and Gre-like transcription factors. The mycobacterial HelD NCC-domain tip does not contain the conserved DXX(E/D) motif necessary for Gre factor-like endonuclease activity. Sequence alignment was performed using Clustal Omega[12] and the graphics was created in ESPript 3.0[10].

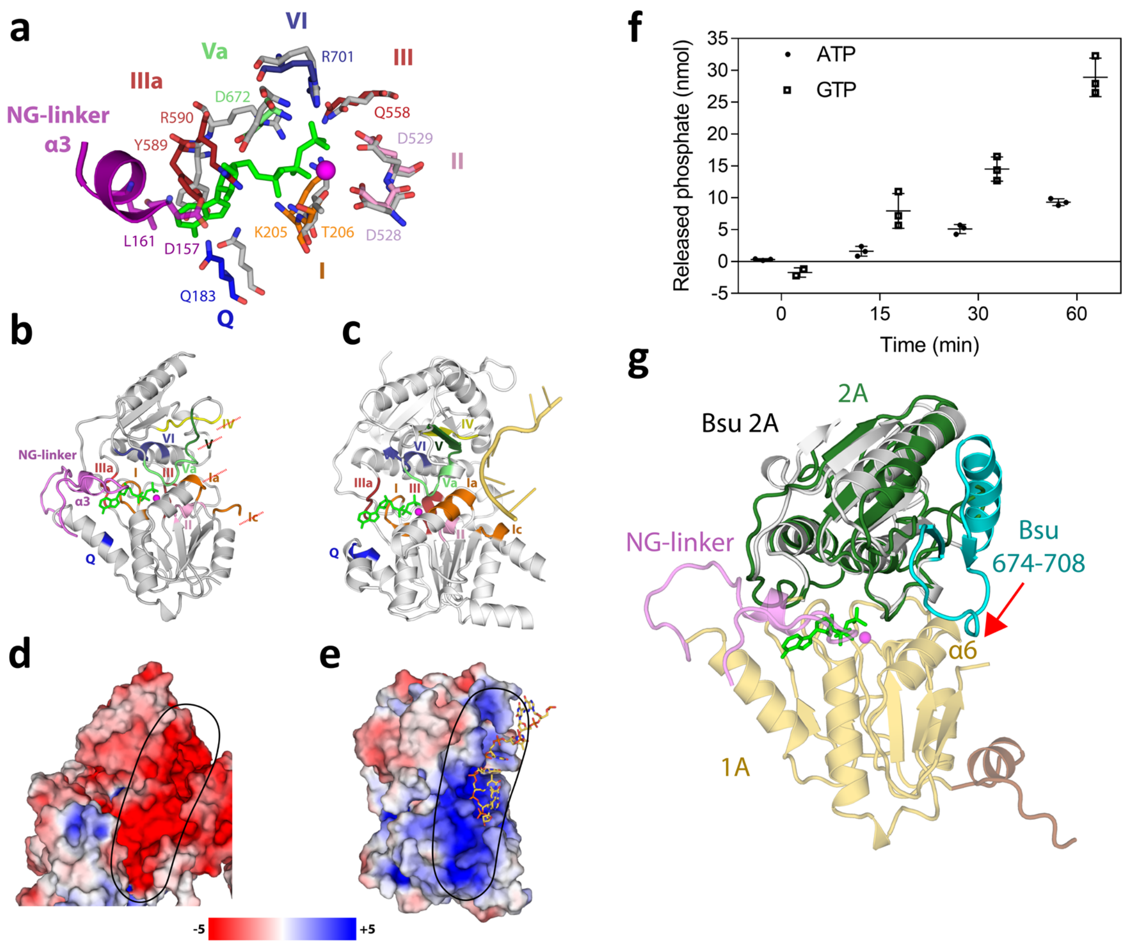

Supplementary Figure 9: *Msm* HelD 1A-2A heterodimer nucleotide binding site compared to UvrD; NTPase activity of *Msm* HelD; *Bsu* HelD CTD crystal structure.

a, Superposition of the HelD NTP-binding site (State I, color coded) and the UvrD ATP-bound state (grey, PDB ID 2IS4). Conserved residues from motifs Q (blue), I (orange), II (pink), ~III and IIIa (firebrick), Va (lightgreen) and VI (deepblue) are present but not in conformations compatible with NTP binding. The ordered NG-linker locks the conformation of Tyr589 (Van der Waals interactions with residues HelD/157, 160 and 161 of α3) and of Arg590 (Arg side chain links Asp157 and Glu672 of HelD) so that they would clash with the NTP base and ribose, probably making the NTP binding/hydrolysis in State I impossible.

b and **c**, Conserved nucleotide binding site motifs Q, I, II, III, IIIa, Va, and VI (color coded as in Figure 2d) as observed in HelD (a, b) in comparison to UvrD [(c),PDB ID 2IS4]. Residues responsible for ssDNA [pale yellow in (c)] binding in motifs Ia and Ic (orange), IV (yellow) and V (forest green) in UvrD are not present in HelD (red crossing).

d and **e**, Comparison of surface electrostatic potential of the HelD 1A-2A heterodimer and UvrD ssDNA-bound 1A-2A heterodimer, respectively. A prominent positively charged groove binds ssDNA (sticks in e) on the surface of UvrD (black oval). In contrast, a negatively charged groove is present in a similar area of HelD surface (black oval). Electrostatics surfaces were generated by APBS[13]within PyMol according to heat bar in k_BT/e units.

f, Hydrolyses of ATP and GTP were monitored and evaluated at 0, 15, 30 and 60 min intervals. Measurements were performed in 3 biological replicates for each time interval with separate background readings for each condition. The results are shown as mean values of the amounts of released phosphate in the reaction, with standard deviations shown as error bars. The symbols are individual replicates (n=3). The data were analysed and the graphics created with GraphPad Prism 7.02.

g, X-ray structure of the C-terminal domain of *Bsu* HelD compared with State I of *Msm* HelD. The C-terminal domain of *Bsu* HelD (residues 608-773) shown as secondary structure elements in grey superimposed by the SSM algorithm with the 2A domain of *Msm* HelD (colored as in Figure 1d); ATP (green sticks) and Mg^{2+} (magenta sphere) in positions as in the structure 2IS4 superimposed according to the NTP-binding site motifs in *Msm* HelD. The 2A domain structure of *Bsu* HelD corresponds to the Rossman fold of the RecA-like domain (central twisted 5-stranded β-sheet surrounded by 5 α-helices 611-620, 645-663, 674-687, 733-745, and 760-764); loop 624-630 was not localized. The domain is most similar to the crystal structure of the C-terminal domain of putative DNA helicase from *Lactobacillus plantarum* (PDB ID 3DMN, rmsd 1.23 Å, 151 aligned C^{α} atoms, 37.7% sequence identity) with identical fold and topology (PDBeFold server[14]). The structure aligns well with that of the 2A/2B domain of UvrD (PDB ID 2IS4, rmsd 1.6 Å, 149 aligned C^{α} atoms), with an almost perfect match of the secondary structure, however of significantly different topology (not shown). The C-terminal domain of *Bsu* HelD has a very similar localization of the amino acid residues forming the expected NTP-binding site (Arg608 corresponds to UvrD/Arg284 – part of motif IIIa, motif VI occurs as 741-TACTRAM-747, Arg745 very likely participating in NTP binding and cleavage, Glu716 is conserved in position of UvrD/Glu566, likely binding the NTP ribose moiety). In comparison with State I of *Msm* HelD the *Bsu* structure is more similar to the 2A domain (rmsd 2.2 Å, 92 aligned residues, sequence identity of the aligned parts 21.7%, alignment shown) than to 1A (2.7 Å, 102 residues aligned, 9.8%, alignment not shown). The helix-loop-strand motif 674-708 (cyan) of *Bsu* HelD does not match any element of 2A in *Msm* HelD and the region 695-699 of the loop would clash (red arrow) with α6 of domain 1A in *Msm* HelD.

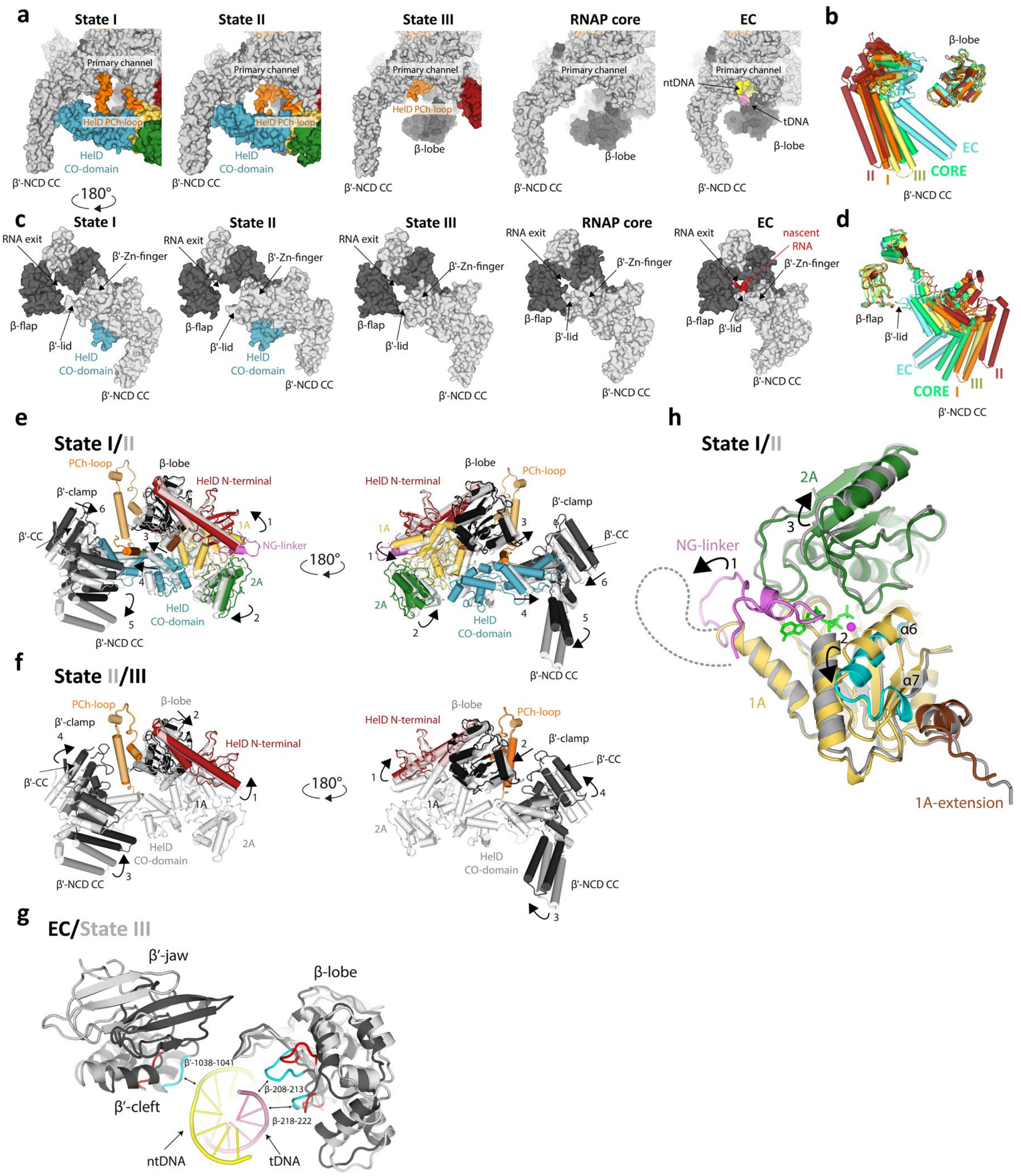

Supplementary Figure 10: The *Msm* HelD specific domain wedges into the RNAP primary channel; global domain changes in *Msm* HelD states.

a, Surface representation of HelD specific domain interaction with RNAP primary channel in State I, II, and III, compared to *Msm* RNAP core (PDB ID 6F6W) and model of *Msm* elongation complex according to PDB ID 2O5J. Color code as in Figure 1d, template DNA in pink, non-template in yellow.

b, Comparison of RNAP primary channel opening in RNAP complex with HelD in State I (orange), II (red), III (yellow), and without HelD in RNAP core (green) in EC (cyan).

c, Surface representation of RNA exit channel opening caused by HelD interaction with RNAP in State I, II, and III, compared to *Msm* RNAP core (PDB ID 6F6W) and model of *Msm* elongation complex according to PDB ID 2O5J. Color code as in Figure 1d, nascent RNA in red.

d, Comparison of RNAP RNA exit channel opening in RNAP complex with HelD in State I (orange), II (red), III (yellow), and without HelD in RNAP core (green) in EC (cyan).

e, Two views of State I and II superposition according to the RNAP core (β/430-738). The collapse of NG-linker in State II allows for 1A and 2A mutual reorientation (arrow 1 and 2). Concomitantly this causes a shift of 1A extension (arrow 3 in left panel) and β-lobe (arrow 3 in right panel). The reorientation of 1A-2A also causes a shift of the HelD CO-domain (arrow 4) and a further swing-out of β'-NCD CC (arrow 5). On the other hand the β'-CC shifts towards the HelD CO-domain (arrow 6). State I is colored as in Figure 1, State II is in light transparent grey. Only selected domains are displayed.

f, Two views of State II and III superposition according to the RNAP core (β/430-738). In State III, the HelD N-terminal domain slightly shifts within the RNAP secondary channel (arrow 1). The absence of 1A and 2A domains in State III allows relaxation of β-lobe, which shifts to a similar position as in State I (arrow 2), The absence of the HelD-specific domain allows closure of the β'-clamp (arrow 3 and 4). State III is colored as in Figure 1; State II is in light transparent grey as in (e). Only selected domains are displayed.

g, Superposition of State III (grey) with EC (black) according to the RNAP core (β/430-738), only selected domains are displayed. The HelD N-terminal domain insertion into the secondary channel induces changes in the RNAP primary channel that may destabilise the dwDNA interaction. Notice the shifts of both β-lobe and β'-jaw/cleft and changes in the loops contacting (double arrows) dwDNA in EC (cyan) and in the HelD presence (red).

h, Superposition of the 1A-2A heterodimer in State I (colored as in Figure 1) and State II (light grey) according to 1A-1 domain (1A-1 residues 174-259 superimposed by least squares on main chain, rmsd 2.37 Å). In state II, the disorder of NG-linker (arrow 1), rearrangement of α6 and formation of α7 (change from yellow to cyan, arrow 2), and shift of the 2A domain (arrow 3) altogether result in more open NTP-binding site (ATP in green, Mg^{2+} magenta sphere, modelled by superposition with UvrD ternary complex, PDB ID 2IS4).

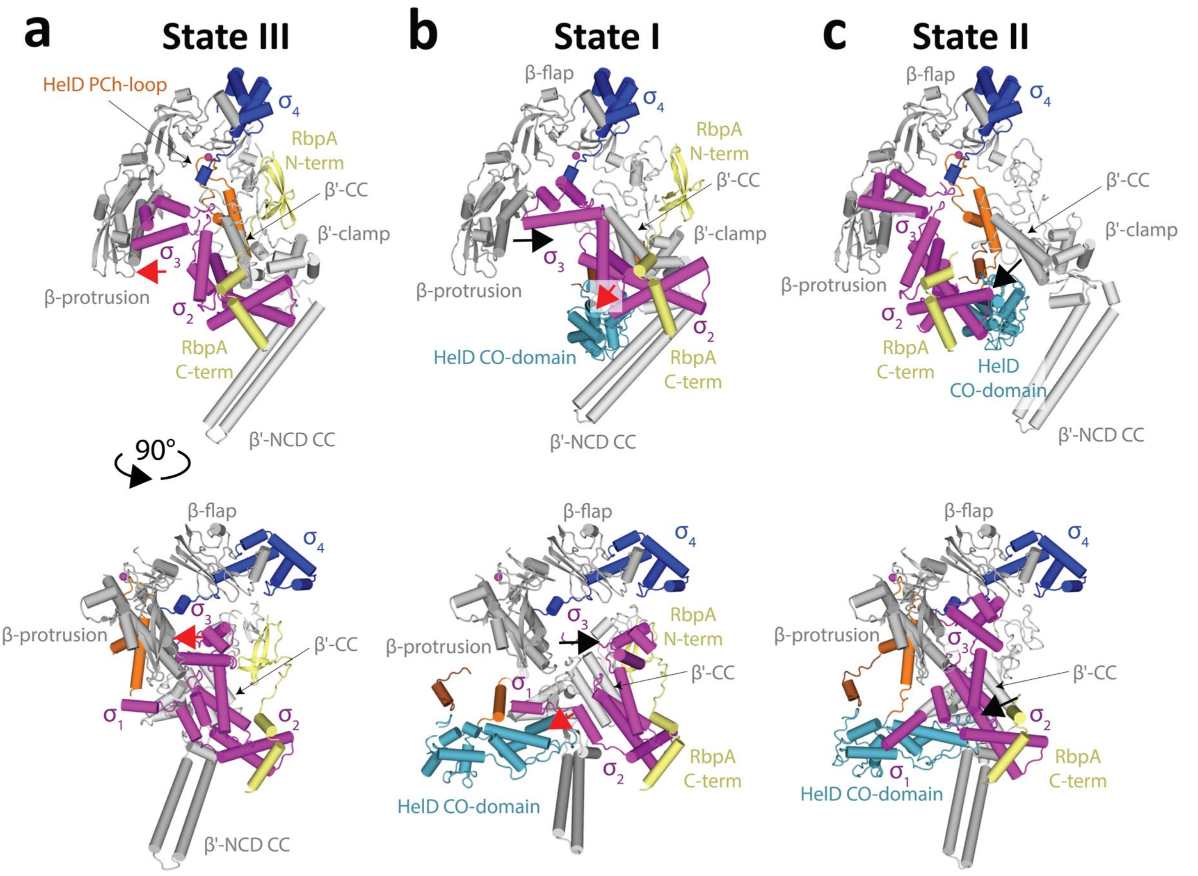

Supplementary Figure 11: Models of HelD, σ^A, and RbpA coexistence on RNAP.

a,**b**,**c** Three hypothetical coexistence modes (ordered according to the least adjustments needed) of HelD (only HelD-specific domains shown for clarity), σ^A, and RbpA in the RNAP primary channel in two perpendicular views. Color code as in Figure 1, domains σ^{1-3} in magenta, σ^4 in blue, RbpA in yellow.

a, The State III complex superimposed with PDB entries ID 6EYD and ID 5TW1 based on the RNAP core domain (β/430-738). In State III, the HelD CO-domain does not occupy the primary channel, and σ^2 can interact with the conserved binding site on the β'-clamp coiled-coil domain (β'-CC). The σ^3 domain clashes sterically with β-protrusion (also called β-domain 1, red arrow), however, a slight shift of σ^3 could accommodate the latter. The RbpA interaction with both σ^A and β'-clamp is preserved.

b, The State I complex superimposed with the PDB entries ID 6EYD and ID 5TW1 based on the RNAP β'-clamp (β'/6-404). In State I, the HelD CO-domain occupies the primary channel and σ^2 can interact with β'-CC if the CO-tip accommodates for σ^2 presence (red arrow) and σ^1 moves away. The opening of the RNAP clamp in State I causes σ^3 detachment from domain 1 (black arrow). The protein linker between σ^3 and σ^4 has to accommodate the RNAP clamp opening. The RbpA interaction with both σ^A and β'-clamp is preserved.

c, The State II complex superimposed with the PDB entry ID 5TW1 based on the RNAP core domain (β/430-738). In State II, the HelD CO-domain occupies the primary channel and moves

even further towards β'-CC, disallowing σ^2 to bind the β'-clamp. In this situation σ^3 and σ^4 hold only on the β-protrusion and β-flap and σ^2 detaches from the β'-clamp (black arrow). The resulting gap between σ^2 and β'-clamp may be filled with the HelD CO-domain.

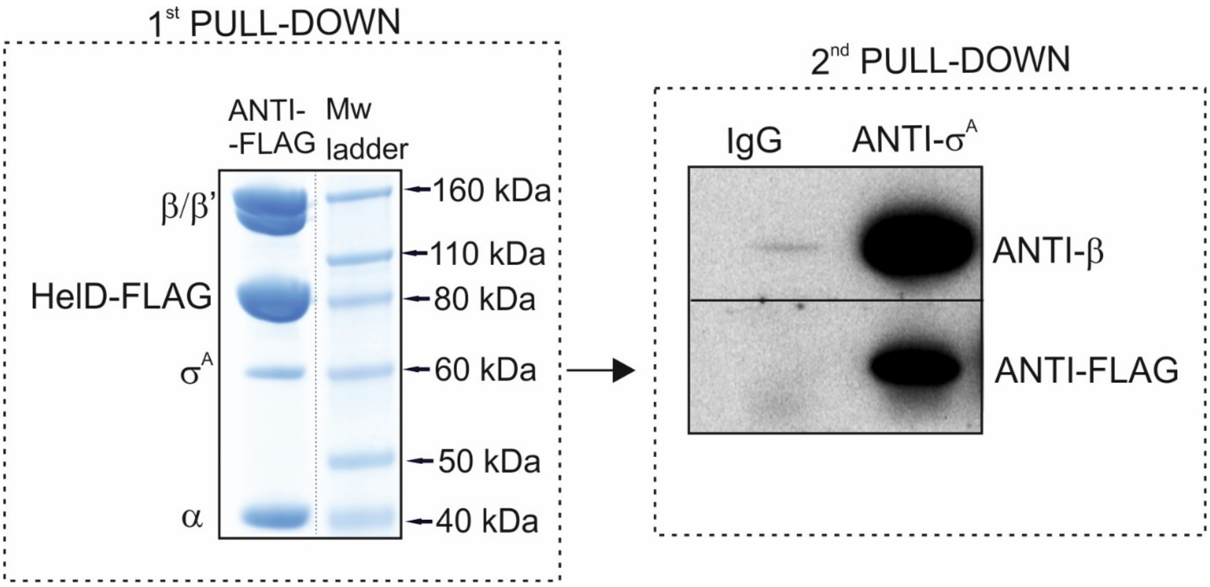

Supplementary Figure 12: HelD and σ^A can coexist on RNAP.

Double pull-down: The first pull-down was performed from *Msm* lysates (strain LK2590) with an antibody against the FLAG peptide (the same result as in Figure 4f). The Simply Blue-stained gel shows the resulting pulled-down proteins – first lane (ANTI-FLAG). The second lane shows Molecular weight (Mw) ladder. The two lanes were assembled electronically – marked with the dotted line. The protein mixture from the first pull-down was then used for the second pull-down with an antibody against σ^A and with IgG (negative control). The presence of HelD-FLAG (anti-FLAG) and RNAP (anti-β) was verified by Western blotting. The identities of the antibodies used for the detection are indicated next to the gel. The experiment was performed 2x with identical results.

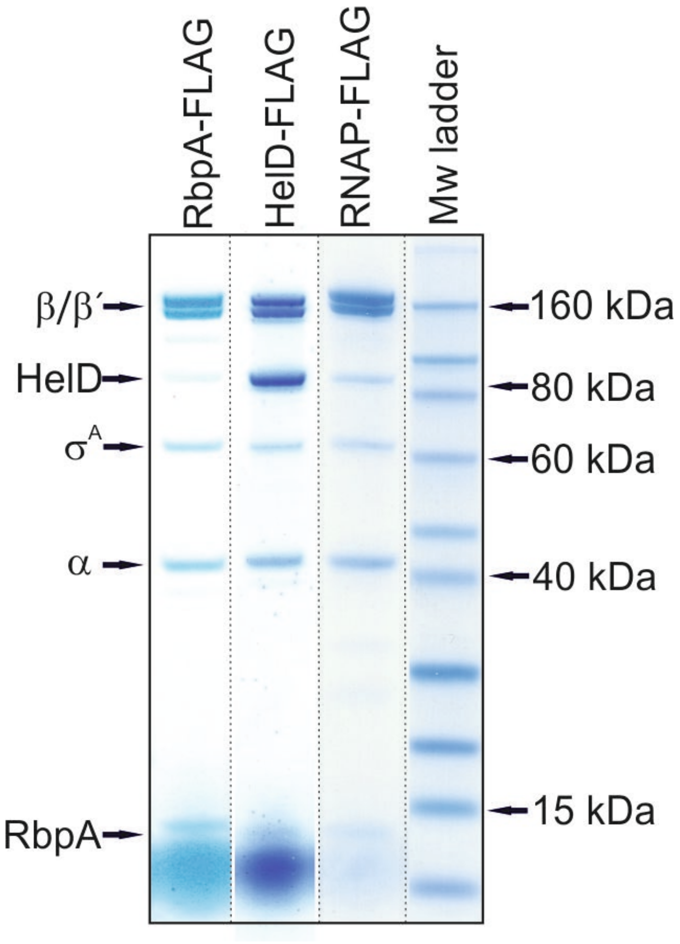

Supplementary Figure 13: RbpA is in complex with RNAP, σ^A, and HelD.

Simply Blue-stained SDS-PAGE of IPs of FLAG-tagged proteins from *Msm* (RbpA-FLAG, strain LK2541; HelD-FLAG, strain LK2590; RNAP-FLAG, strain LK1468). The identities of the FLAG-tagged proteins are indicated above the lanes. The identities of the pulled-down proteins are indicated with arrows (determined by mass spectrometry). The final gel was assembled electronically as indicated with the dotted lines. The experiment was performed 3x (biological replicates) with identical results.

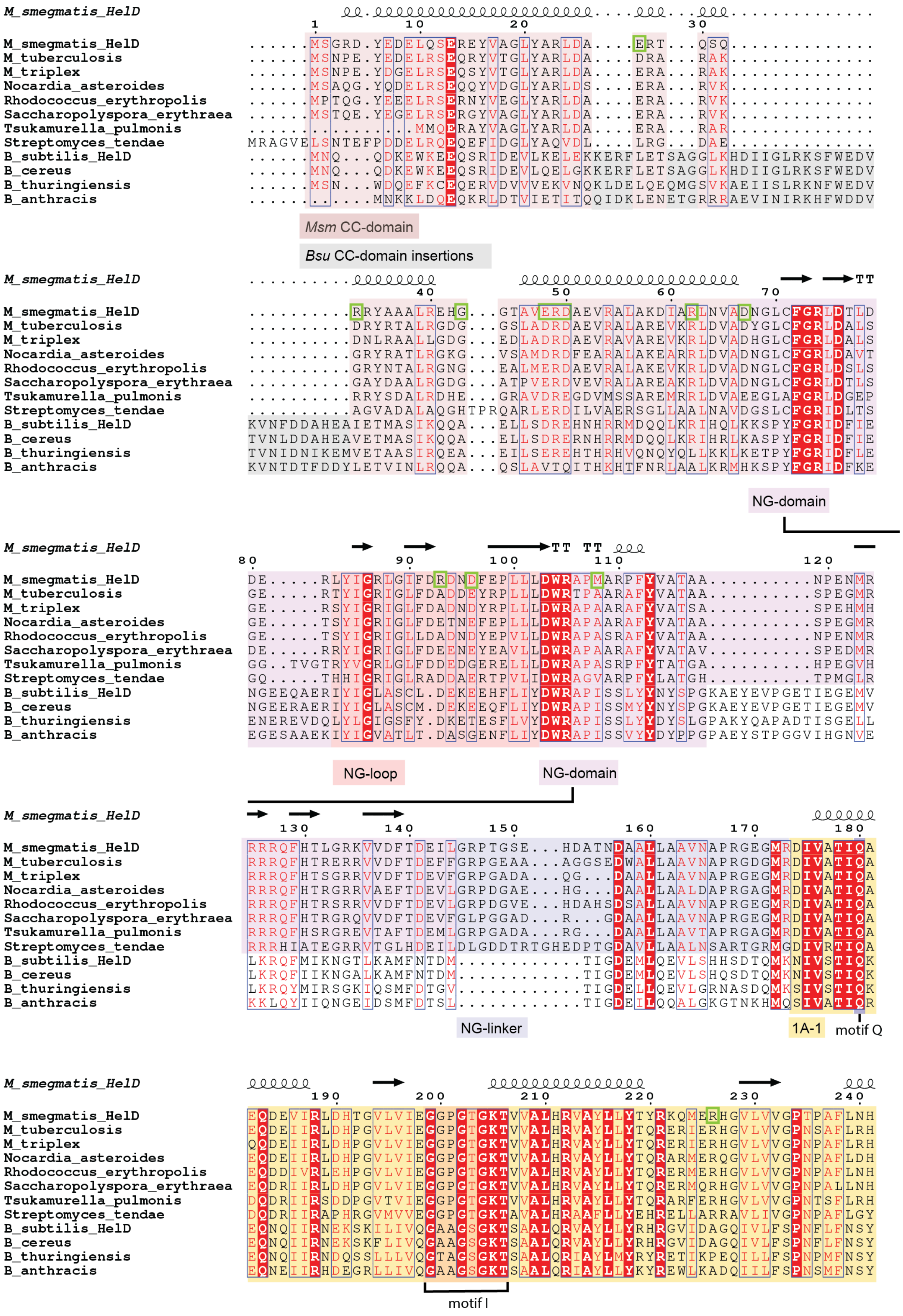
M_smegmatis_HelD
M_tuberculosis
M_triplex
Nocardia_asteroides
Rhodococcus_erythropolis
Saccharopolyspora_erythraea
Tsukamurella_pulmonis
Streptomyces_tendae
B_subtilis_HelD
B_cereus
B_thuringiensis
B_anthracis
Msm CC-domain
Bsu CC-domain insertions
NG-domain
NG-loop
NG-domain
NG-linker
1A-1
motif Q
motif I

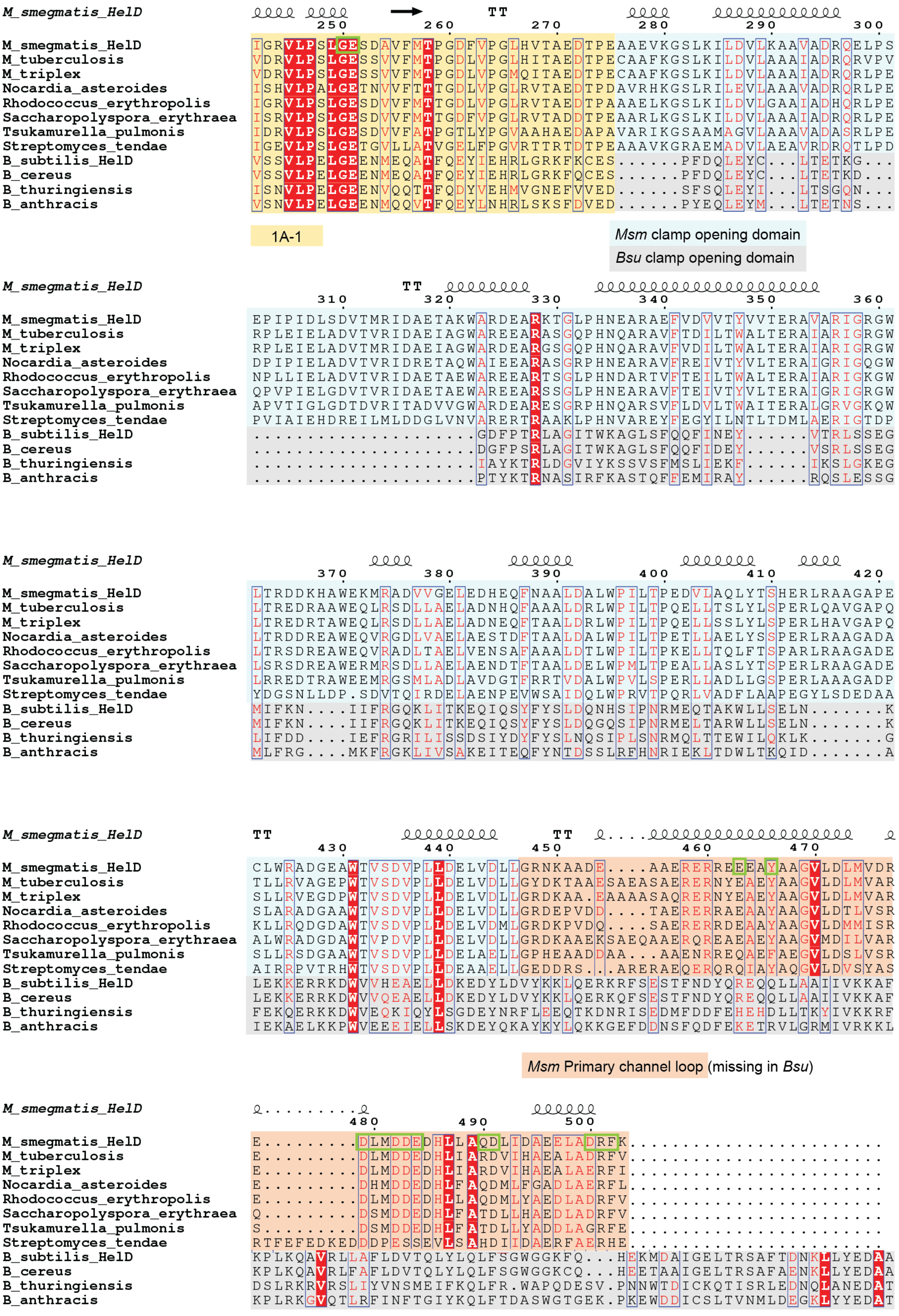
M_smegmatis_HelD
M_tuberculosis
M_triplex
Nocardia_asteroides
Rhodococcus_erythropolis
Saccharopolyspora_erythraea
Tsukamurella_pulmonis
Streptomyces_tendae
B_subtilis_HelD
B_cereus
B_thuringiensis
B_anthracis
1A-1
Msm clamp opening domain
Bsu clamp opening domain
Msm Primary channel loop (missing in Bsu)

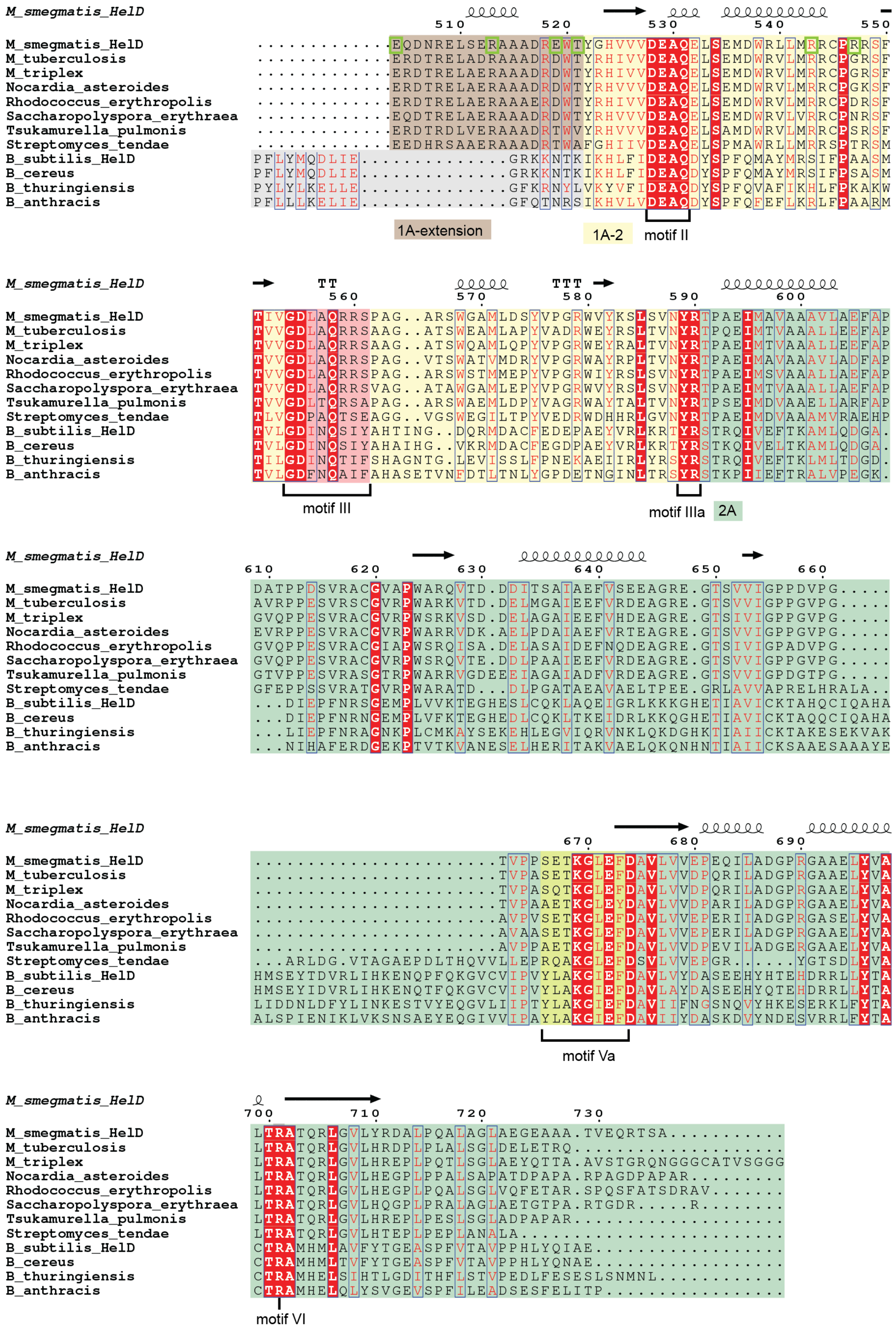
1A-extension
1A-2
motif II
motif III
motif IIIa
2A
motif Va
motif VI

Supplementary Figure 14: Sequence alignment of HelD homologs.

Curated sequence alignment based on alignment generated by Clustal Omega software[12]. Amino acids in *Msm* HelD that make contacts with the RNAP core as observed in State II (Supplementary Tables 1-3) are marked with green rectangles. Secondary structure is denoted for *M. smegmatis* HelD. GeneBank codes of used sequences: *Msm* WP_003893549.1, *M. tuberculosis*: PLV44927.1; *M. triplex*: CDO88184.1, *Nocardia asteroides*: GAD85771.1, *Rhodococcus erythropolis*: WP_095971734.1, *Saccharopolyspora erythraea*: PFG97077.1, *Tsukamurella pulmonis*: WP_139061895.1; *Streptomyces tendae*: WP_150152972.1, *Bsu* WP_003244180.1, *Bacillus cereus* WP_095971734.1, *B. thuringiensis*: WP_074790911.1, *B. anthracis*: WP_071737252.1. The graphics was created using ESPript 3.0[15]

Supplementary Table 1: Hydrogen bonds and salt bridges between HelD N-terminal domain (State II) and RNAP β' subunit.

Interactions up to 4 Å distance according to the PDBe PISA server[14].

#	RNAP β' subunit	HelD residue
1	D:LYS 775	H:GLU 27
2	D:ASN 809	H:GLY 43
3	D:LYS 820	H:GLU 48
4	D:ARG 865	H:ASP 50
5	D:ARG 757	H:ASP 96
6	D:GLN 778	H:ARG 34
7	D:GLN1008	H:ARG 49
8	D:GLN1146	H:ARG 49
9	D:GLU 751	H:ARG 93
10	D:ASP 779	H:ARG 93
11	D:GLY 762	H:MET 108
12	D:ARG 865	H:ASP 50
13	D:ARG1086	H:ASP 67
11	D:GLU 771	H:ARG 62

Supplementary Table 2: Hydrogen bonds and salt bridges between HelD 1A domain (State II) and RNAP β-lobe and β'-jaw.

Interactions up to 4 Å distance according to the PDBe PISA server[14].

#	RNAP β subunit	HelD residue
1	C:LYS 188	H:THR 521
2	C:SER 185	H:ARG 513
3	C:GLU 187	H:ARG 226
4	C:GLU 187	H:ARG 513
5	C:LYS 209	H:GLU 519
6	C:ARG 210	H:GLU 519
7	C:ARG 210	H:ARG 543
8	C:LYS 209	H:THR 521
9	C:ASP 211	H:ARG 547
	RNAP β' subunit	
1	D:VAL1040	H:GLU 504
2	D:LYS1061	H:GLY 250
3	D:ARG1084	H:GLU 251

Supplementary Table 3: Hydrogen bonds and salt bridges between HelD primary channel loop (State I and II) and RNAP β and β′ constituents of the primary channel.

Interactions up to 4 Å distance according to the PDBe PISA server[14].

**State I**		
#	RNAP β′ subunit	HelD residue
1	D:ARG1205	H:ALA 467
**State II**		
#	RNAP β subunit	HelD residue
1	C:LYS 184	H:ASP 500
2	C:ARG 456	H:GLN 490
3	C:ARG 464	H:ASP 491
4	C:GLN 605	H:GLU 484
5	C:LYS 875	H:ASP 483
6	C:LYS 883	H:ASP 483
7	C:HIS1026	H:GLU 484
8	C:HIS1026	H:GLU 484
9	C:ARG1058	H:ASP 479
	RNAP β′ subunit	
1	D:TYR 871	H:GLU 463
2	D:ARG 875	H:GLU 463
3	D:ARG 874	H:TYR 466
4	D:ARG 427	H:ASP 479
5	D:ARG 421	H:ASP 479
6	D:ARG 427	H:LEU 480
7	D:ARG 500	H:MET 481
8	D:GLN 540	H:MET 481
9	D:ALA 542	H:MET 481
10	D:ARG 500	H:ASP 482
11	D:ARG1039	H:PHE 502
12	D:ARG 874	H:TYR 466
13	D:ASP 878	H:TYR 466
14	D:ASP 539	H:ASP 483
15	D:ARG1012	H:ARG 501
16	D:ASP 868	H:ARG 501

Supplementary Table 4: Bacterial strains.

	Strain	Description/Notes	Source
E. coli			
RNAP *Msm*	LK1853		[16]
SigA(σ^A) *Msm*	LK1740	pET22b+ with C-terminal 6xHis SigA *Msm* BL21(DE3)	This work
HelD *Msm*	Mshe1	6xHis-HelD *Msm*, Lemo21 (DE3)	This work
RbpA *Msm*	LK1254	pET22b+ with C-terminal 6xHis RbpA *Msm*, BL21(DE3)	This work
M. smegmatis			
wt	LK865	*M. smegmatis* mc^2 155	Laboratory strain
RNAP-FLAG	LK1468 MR-sspB	kindly provided by D. Schnappinger, Weill Cornell Medical College, New York, USA	[17]
RbpA-FLAG	LK2541		This work
SigA-FLAG	LK2073		This work
HelD-FLAG	LK2590		This work

Supplementary Table 5: DNA oligonucleotides.

Primer	Sequence *5'→ 3'*	
#1101	AAATCGGGCGGCGTCCCGGA	Primers for *Msm* DNA fragment for EMSA assays
#1146	ACGGAAGCTTGGCGAGGC	
#1155	GGAATTCCATATGGTGGCAGCGACAAAGGCA	Primers for σ^{A} (*MSMEG_2758*) cloning into pET22b
#1156	CCGCTCGAG GTCCAGGTAGTCGCGCAG	
#1182	CCGCTCGAGGCTTCCGGTTCCGCGCCG	Primers for *rbpA* (*MSMEG_3858*) cloning into pET22b
#1183	GGAATTCCATATGATGGCTGATCGTGTCCTG	
#2339	CTTCATATGGCAGCGACAAAGGCAAGCCCG	Primers for σ^{A} (*MSMEG_2758*) cloning into pTet-Int
#2340	CGTAAGCTTCTACTTGTCGTCGTCGTCCTTGTAGTCCAGGTAGTCGCGCAGCAC	
#2894	ATTCCATATGGCTGATCGTGTCCTGCGGGGC	Primers for *rbpA* (*MSMEG_3858*) cloning into pTet-Int
#3093	CGTAAGCTTCTACTTGTCGTCGTCGTCCTTGTAGTCGCTTCCGGTTCCGCGCCGCTT	
#3130	CATTCATATGTCAGGTCGGGACTACGAGGAC	Primers for *helD* (*MSMEG_2174*) cloning into pTetInt
#3131	CGTAAGCTTCTACTTGTCGTCGTCGTCCTTGTAGTCTGCCGACGTGCGCTGCTCGACCGT	

Supplementary Table 6. Cryo-EM data collection, refinement and validation statistics.

	Msm HelD-RNAP complex **State I**	*Msm* HelD-RNAP complex **State II**	*Msm* HelD-RNAP complex **State III**
**Deposition**	EMD-10996, PDB ID 6YXU	EMD-11004, PDB ID 6YYS	EMD-11026, PDB ID 6Z11
**Data collection and processing**			
Magnification	165,000	165,000	165,000
Voltage (kV)	300	300	300
Electron exposure ($e^-/Å^2$)	40-50	40-50	40-50
Defocus range (μm)	0.7-3.3	0.7-3.3	0.7-3.3
Pixel size (Å)	0.8311	0.8311	0.8311
Symmetry imposed	C1	C1	C1
Initial particle images (no.)	1,560,500	1,560,500	1,560,500
Final particle images (no.)	185,400	173,500	119,100
Map resolution (Å)	3.08	3.08	3.47
FSC threshold	0.143	0.143	0.143
Map resolution range (Å)	3.08-5.90	3.02-5.90	3.29-5.90
Estimated angular accuracy (°)	0.693	0.729	0.795
Efficiency score[2]	0.4	0.50	0.65
Sphericity[9]	0.938	0.919	0.916
**Refinement**			
Initial model used (PDB code)	6F6W[11]	6F6W	6F6W
Model resolution (Å)	3.2	3.2	3.5
FSC threshold	0.5	0.5	0.5
Model resolution range (Å)	3.09-5.90	3.02-5.90	3.05-5.90
Map sharpening *B* factor ($Å^2$)	-78.53	-81.37	-85.45
Model vs map cross correlation	0.81	0.79	0.81
Model composition			
Non-hydrogen atoms	27791	27930	23948
Protein residues	3583	3597	3077
Nucleotide residues	0	0	0
Ligands	3	3	3
B factors ($Å^2$)			
Protein	40.27	32.39	34.47
Ligand	61.69	47.49	46.56
R.m.s. deviations from ideal			
Bond lengths (Å)	0.006	0.005	0.005
Bond angles (°)	0.672	0.656	0.610
Validation			
MolProbity score	2.03	2.00	2.01
Clashscore	9.28	9.18	7.94
Poor rotamers (%)	0.00	0.00	0.04
Ramachandran plot			
Favored (%)	90.54	91.15	89.03
Allowed (%)	9.43	8.82	10.97
Disallowed (%)	0.03	0.03	0

Supplementary Table 7: Data collection and refinement statistic of the *B. subtilis* HelD C-terminal domain. Values in parentheses refer to the highest resolution shell.

PDB code	6VSX
**Data collection**	
X-ray source	Rigaku MicroMax 007 HF
Wavelength (Å)	1.54178
No. if oscillation images	1080
Total oscillation angle	1080
$\Delta\phi$ (°)	1
Crystal to detector distance (mm)	50
Average mosaicity (°)	1.4
Space group	$C2_1$
Cell dimensions	
a (Å)	106.96
b (Å)	38.81
c (Å)	44.43
β (º)	101.45
Resolution (Å)	25.0 – 2.0
No. of all observed reflections	245,968
No. of unique reflections	11,905
Average redundancy	20.7 (14.1)
Completeness (%)	96.7 (72.0)
$I/\sigma(I)$	60.1 (14.3)
Wilson B-factor (Å^2)	21.87
R-merge	0.044 (0.206)
CC1/2	(0.991)
CC*	(0.998)
**SAD Phasing (S and P)**	
Number of sites	10 (S) and 1 (P)
Figure of Merit	0.296
**Refinement**	
Resolution (Å)	25.0 – 2.0
No. of reflections used in refinement	11,869 (1,186)
R_{work}	0.1723 (0.1756)
R_{free}	0.2014 (0.2393)
No. of atoms	1,382
macromolecules	1,268
ligands	5
solvent	109
No. of protein residues	159
RMS deviations from ideal	
bond lengths (Å)	0.007
bond angles (°)	0.80
Clashscore (Molprobity)	5.92
Ramachandran plot, residues	
in favored region (%)	98.06
outliers (%)	0.0
Average B-factor (Å^2)	25.2
Macromolecules (Å^2)	24.6
Ligands (Å^2)	30.5
Solvent (Å^2)	32.9

Supplementary References

1 Zheng, S. Q. *et al.* MotionCor2: anisotropic correction of beam-induced motion for improved cryo-electron microscopy. *Nat Methods* **14**, 331-332, doi:10.1038/nmeth.4193 (2017).

2 Naydenova, K. & Russo, C. J. Measuring the effects of particle orientation to improve the efficiency of electron cryomicroscopy. *Nat Commun* **8**, 629, doi:10.1038/s41467-017-00782-3 (2017).

3 Rosenthal, P. B. & Henderson, R. Optimal determination of particle orientation, absolute hand, and contrast loss in single-particle electron cryomicroscopy. *J Mol Biol* **333**, 721-745 (2003).

4 Jakobi, A. J., Wilmanns, M. & Sachse, C. Model-based local density sharpening of cryo-EM maps. *Elife* **6**, doi:10.7554/eLife.27131 (2017).

5 Zivanov, J. *et al.* New tools for automated high-resolution cryo-EM structure determination in RELION-3. *Elife* **7**, doi:10.7554/eLife.42166 (2018).

6 Zivanov, J., Nakane, T. & Scheres, S. H. W. Estimation of high-order aberrations and anisotropic magnification from cryo-EM data sets in RELION-3.1. *IUCrJ* **7**, 253-267, doi:10.1107/S2052252520000081 (2020).

7 Pettersen, E. F. *et al.* UCSF Chimera--a visualization system for exploratory research and analysis. *J Comput Chem* **25**, 1605-1612, doi:10.1002/jcc.20084 (2004).

8 McNicholas, S., Potterton, E., Wilson, K. S. & Noble, M. E. Presenting your structures: the CCP4mg molecular-graphics software. *Acta Crystallogr D Biol Crystallogr* **67**, 386-394, doi:10.1107/S0907444911007281 (2011).

9 Tan, Y. Z. *et al.* Addressing preferred specimen orientation in single-particle cryo-EM through tilting. *Nat Methods* **14**, 793-796, doi:10.1038/nmeth.4347 (2017).

10 Robert, X. & Gouet, P. Deciphering key features in protein structures with the new ENDscript server. *Nucleic Acids Res* **42**, W320-324, doi:10.1093/nar/gku316 (2014).

11 Laskowski, R. A., Jablonska, J., Pravda, L., Varekova, R. S. & Thornton, J. M. PDBsum: Structural summaries of PDB entries. *Protein Sci* **27**, 129-134, doi:10.1002/pro.3289 (2018).

12 Madeira, F. *et al.* The EMBL-EBI search and sequence analysis tools APIs in 2019. *Nucleic Acids Res* **47**, W636-W641, doi:10.1093/nar/gkz268 (2019).

13 Jurrus, E. *et al.* Improvements to the APBS biomolecular solvation software suite. *Protein Sci* **27**, 112-128, doi:10.1002/pro.3280 (2018).

14 Krissinel, E. & Henrick, K. Inference of macromolecular assemblies from crystalline state. *J Mol Biol* **372**, 774-797, doi:10.1016/j.jmb.2007.05.022 (2007).

15 Le, T. T. *et al.* Mfd Dynamically Regulates Transcription via a Release and Catch-Up Mechanism. *Cell* **172**, 344-357 e315, doi:10.1016/j.cell.2017.11.017 (2018).

16 Kouba, T. *et al.* The Core and Holoenzyme Forms of RNA Polymerase from Mycobacterium smegmatis. *J Bacteriol* **201**, doi:10.1128/JB.00583-18 (2019).

17 Kim, J. H. *et al.* Protein inactivation in mycobacteria by controlled proteolysis and its application to deplete the beta subunit of RNA polymerase. *Nucleic Acids Res* **39**, 2210-2220, doi:10.1093/nar/gkq1149 (2011).
