# Supplementary material for: Mycobacterial HelD is a nucleic acids-clearing factor for RNA polymerase
Source: Nat Commun. 2020 Dec 18;11:6419. doi: 10.1038/s41467-020-20158-4 (PMC7749160; doi:10.1038/s41467-020-20158-4)
Supplement: Supplementary file 3 — Description of Additional Supplementary Files [file 41467_2020_20158_MOESM3_ESM.pdf]

Description of Additional Supplementary Files

Supplementary Movie 1: Cryo-EM structure of the *Msm* HelD-RNAP complex in State I – PCh-engaged. The movie shows LocScale filtered cryo-EM map of State I in two perpendicular rotations. The individual RNAP parts and HelD domains are labelled and color-coded as in Fig. 1.

Supplementary Movie 2: Cryo-EM structure of the *Msm* HelD-RNAP complex in State II – PCh-engaged and AS-interfering. The movie shows LocScale filtered cryo-EM map of State II in two perpendicular rotations. The individual RNAP parts and HelD domains are labelled and color-coded as in Fig. 1.

Supplementary Movie 3: Cryo-EM structure of the *Msm* HelD-RNAP complex in State III – PCh dis-engaged and AS-interfering. The movie shows LocScale filtered cryo-EM map of State III in two perpendicular rotations. The individual RNAP parts and HelD domains are labelled and color-coded as in Fig. 1.
