# Supplementary material for: Mycobacterial HelD is a nucleic acids-clearing factor for RNA polymerase
Source: Nat Commun. 2020 Dec 18;11:6419. doi: 10.1038/s41467-020-20158-4 (PMC7749160; doi:10.1038/s41467-020-20158-4)
Supplement: Supplementary file 7 — Reporting summary [file 41467_2020_20158_MOESM7_ESM.pdf]

nature research

Corresponding author(s): Tomas Kouba, Jan Dohnalek, Libor Krasny

Last updated by author(s): Nov 2, 2020

Reporting Summary

Nature Research wishes to improve the reproducibility of the work that we publish. This form provides structure for consistency and transparency in reporting. For further information on Nature Research policies, see our Editorial Policies and the Editorial Policy Checklist.

Statistics

For all statistical analyses, confirm that the following items are present in the figure legend, table legend, main text, or Methods section.

n/a	Confirmed	
☐	☒	The exact sample size (*n*) for each experimental group/condition, given as a discrete number and unit of measurement
☐	☒	A statement on whether measurements were taken from distinct samples or whether the same sample was measured repeatedly
☐	☒	The statistical test(s) used AND whether they are one- or two-sided<br>*Only common tests should be described solely by name; describe more complex techniques in the Methods section.*
☒	☐	A description of all covariates tested
☒	☐	A description of any assumptions or corrections, such as tests of normality and adjustment for multiple comparisons
☐	☒	A full description of the statistical parameters including central tendency (e.g. means) or other basic estimates (e.g. regression coefficient) AND variation (e.g. standard deviation) or associated estimates of uncertainty (e.g. confidence intervals)
☐	☒	For null hypothesis testing, the test statistic (e.g. *F*, *t*, *r*) with confidence intervals, effect sizes, degrees of freedom and *P* value noted<br>*Give P values as exact values whenever suitable.*
☒	☐	For Bayesian analysis, information on the choice of priors and Markov chain Monte Carlo settings
☒	☐	For hierarchical and complex designs, identification of the appropriate level for tests and full reporting of outcomes
☒	☐	Estimates of effect sizes (e.g. Cohen's *d*, Pearson's *r*), indicating how they were calculated

Our web collection on statistics for biologists contains articles on many of the points above.

Software and code

Policy information about availability of computer code

Data collection	EPU 2.5
Data analysis	MotionCor2 v1.3.1, RELION 3.0, RELION 3.1, WARP 1.0.5, Gautomatch 0.56, CCP-EM v1.4.1, LocScale 0.1, Molrep 11.7.02, Coot 8.9.1, Coot 8.9.2, Buccaneer 1.6.5, MAINMAST 1.0, Phenix.refine 1.16_3549, Phenix program suite 1.18.1, PyMOL 1.9, PyMOL 2.0.6, APBS 1.5, USCF Chimera 1.11.2, CCP4MG 2.10.11, PDBePISA 1.48, HKL2000 712, AutoSol 1.18.1, AutoBuild 1.18.1, GraphPad Prism 7.02, Quantity One software 4.6.3 (Bio-Rad), Clustal Omega 1.2.2, ESPript 3.0, Gctf 1.18, DataAnalysis 5.0, MASCOT v.2.6, 3DFSC 3.0

For manuscripts utilizing custom algorithms or software that are central to the research but not yet described in published literature, software must be made available to editors and reviewers. We strongly encourage code deposition in a community repository (e.g. GitHub). See the Nature Research guidelines for submitting code & software for further information.

Data

Policy information about availability of data

All manuscripts must include a data availability statement. This statement should provide the following information, where applicable:

- Accession codes, unique identifiers, or web links for publicly available datasets
- A list of figures that have associated raw data
- A description of any restrictions on data availability

Co-ordinates and structure factors or maps have been deposited in the wwwPDB or EMDB databases.
Bsu HelD C-terminal domain (X-ray) PDB ID 6VSX (https://www.rcsb.org/structure/6VSX)
Msm HelD-RNAP complex State I (cryoEM) EMD-10996, PDB ID 6YXU (https://www.rcsb.org/structure/6YXU)
Msm HelD-RNAP complex State II (cryoEM) EMD-11004, PDB ID 6YYS (https://www.rcsb.org/structure/6YYS)
Msm HelD-RNAP complex State III (cryoEM) EMD-11026, PDB ID 6Z11 (https://www.rcsb.org/structure/6Z11)

Field-specific reporting

Please select the one below that is the best fit for your research. If you are not sure, read the appropriate sections before making your selection.

☒ Life sciences ☐ Behavioural & social sciences ☐ Ecological, evolutionary & environmental sciences

For a reference copy of the document with all sections, see nature.com/documents/nr-reporting-summary-flat.pdf

Life sciences study design

All studies must disclose on these points even when the disclosure is negative.

Sample size	For biochemical analyses, sample sizes of at least of 3 were chosen (biological replicates) to provide significant results.
Data exclusions	No data were excluded.
Replication	Experiments were performed at least 3x (biological replicates). Reported data show individual replicates, means, and respective SD.
Randomization	This study does not involve subjects that require randomization.
Blinding	This study does not involve subjects that require blinding.

Reporting for specific materials, systems and methods

We require information from authors about some types of materials, experimental systems and methods used in many studies. Here, indicate whether each material, system or method listed is relevant to your study. If you are not sure if a list item applies to your research, read the appropriate section before selecting a response.

Materials & experimental systems

n/a	Involved in the study	
☐	☒	Antibodies
☒	☐	Eukaryotic cell lines
☒	☐	Palaeontology and archaeology
☒	☐	Animals and other organisms
☒	☐	Human research participants
☒	☐	Clinical data
☒	☐	Dual use research of concern

Methods

n/a	Involved in the study	
☒	☐	ChIP-seq
☒	☐	Flow cytometry
☒	☐	MRI-based neuroimaging

Antibodies

Antibodies used

Purified anti-E.coli RNA pol Sigma 70 antibody (clone name 2G10, Biolegend, cat. no. 663208);
Anti-E.coli RNA Polymerase β Antibody (8RB13, Biolegend, cat. no. 663903); RNA polymerase beta antibody (8RB13, GeneTex, cat. no. GTX12087);
ANTI-FLAG (clone M2, Sigma, cat. no. F1804);
Goat-anti-mouse IgG (IR800, Advansta, WesternBright MCF-IR fluorescent Western blotting kit, cat. no. K-12022-010);
Anti-Mouse IgG (whole molecule)-Peroxidase produced in rabbit (Sigma, cat. no. A9044)

Validation

All antibodies were validated by WB analysis. All reacting protein were of the correct Mw. Moreover, wherever possible, we verified the identity of the target proteins also by mass spectrometry.
Data sheets and references:
anti-sigma 70, dilution 1:1,000 (reactivity for E. coli, M. smegmatis https://www.biolegend.com/en-gb/products/purified-anti-e-coli-rna-sigma-70----antibody-18128, Pánek et al, 2011 doi:10.1093/nar/gkq1186);
anti-RNAP β, dilution 1:1,000 (https://www.biolegend.com/en-gb/products/anti-e-coli-rna-polymerase-beta-antibody-10494) and (https://www.genetex.com/Product/Detail/RNA-polymerase-beta-antibody-8RB13/GTX12087; Hnilicová et al, 2014 doi:10.1093/nar/gku793);
anti-FLAG, dilution 1:1,000 (https://www.sigmaaldrich.com/catalog/product/sigma/f1804);
goat-anti-mouse IgG, dilution 1:10,000 (https://advansta.com/products/WesternBright-MCF-application-note);
anti-mouse IgG conjugated with HRP, dilution 1:80,000 (https://www.sigmaaldrich.com/catalog/product/sigma/a9044).
